# Development of Decellularized Fish Skin Scaffold Decorated with Biosynthesized Silver Nanoparticles for Accelerated Burn Wound Healing

**DOI:** 10.1155/2023/8541621

**Published:** 2023-01-31

**Authors:** Surya Prasad Adhikari, Astha Paudel, Anisha Sharma, Baruna Thapa, Neha Khanal, Nisha Shastri, Sourav Rai, Rameshwar Adhikari

**Affiliations:** ^1^Department of Mechanical and Aerospace Engineering, Institute of Engineering, Pulchowk Campus, Tribhuvan University, Lalitpur, Nepal; ^2^College of Biomedical Engineering and Applied Sciences, Purbanchal University, Lalitpur, Nepal; ^3^Centre Department of Chemistry, Tribhuvan University, Kirtipur, Nepal

## Abstract

In this study, decellularized fish skin (DFS) scaffold decorated with silver nanoparticles was prepared for accelerating burn wound healing. The silver nanoparticles (AgNPs) synthesized by the green and facile method using *Aloe vera* leaf at different incubating times were characterized by using X-ray Diffraction (XRD), Fourier Transform Infrared (FT-IR) Spectroscopy, and Ultraviolet-Visible Spectroscopy (UV-Vis spectroscopy). The different characterizations confirmed that the sizes of AgNPs prepared by incubating for 6 hours and 12 hours were 29.1 nm and 35.2 nm, respectively. After that, the different concentrations of the smallest AgNPs were used to dope the DFS scaffold to determine the cell viability. Additionally, an agar well diffusion method was used to screen for antimicrobial activity. Minimum inhibitory concentration (MIC) and minimum bactericidal concentration (MBC) were used to correlate the concentration of AgNPs with its bactericidal effect which was seen from 50 *μ*g/ml. Then, the toxicity with human cells was investigated using a 3-(4, 5-dimethylthiazol-2-yl)-2, 5-diphenyl tetrazolium bromide (MTT) assay with no significant cell viability from the concentration of 50 *μ*g/ml to 200 *μ*g/ml compared to the cocultured and commercial treatments.

## 1. Introduction

Globally, it is reported that 2,65,000 deaths occur each year from fires alone, and over 96% of fatal fire-related burns occur in developing countries including Nepal. In the context of Nepal, burn is the second most common injury in rural areas accounting for 5% of disabilities. The leading cause of death of burn victims is due to infection [1]. In our country, with the lack of biobanks, burns are washed, treated, and covered in gauge bandages instead of the human or porcine skin [2]. The bandage should be changed daily, otherwise the leaching problem can happen [3]. The frequent changing of the bandages would expose the wound to bacteria and microbes, which leads to major disorder and impediment in wound repair. To overcome such problems, it is most necessary for an alternative system to heal burn wounds.

There has been intense investigation to find new natural biomaterials based on decellularized unbroken fish skin scaffold which can be used as the skin substitute in case of a severe burn. The tilapia fish skin has shown a high content of collagen types I and II proteins along with a great tension resistant property and enormous moisture content than human [4]. Moreover, such skin scaffold also has been shown a less healing time and lessening in pain [5].

The numerous nanoparticles were invented and used as a promoter of the wound healing process to improve the healing quality. The nanoparticles, whose surface areas increase exponentially, are the key assets that determine their medical applications. Among different metal nanoparticles, silver (Ag), gold (Au), titanium (Ti), and zinc oxide (ZnO) are the most used and safe contender for wound dressing [6]. Among them, AgNPs are currently used nanoparticles, as therapeutic agents for burn wound healing due to their antimicrobial and anti-inflammatory properties [7]. It is commonly used in the treatment of burns, wound infections, and different ulcers and also to improve infection prevention strategies [8]. Moreover, AgNPs possess features to modulate anti-inflammatory cytokine release in order to promote the wound healing process and stimulate epidermal re-epithelialization through proliferation and relocation of keratinocytes [3, 9]. Furthermore, it forms sulfuric bonds with either bacterial cell membrane proteins or thiol groups of various enzymes, especially those involved in the respiratory chain, and ultimately kills the cell. It can also interfere with deoxyribonucleic acid (DNA) with sulphur and phosphor bonds inhibiting the bacterial multiplication [6, 9, 10].

The fish skin is the envelope of the body that separates and protects the fish from its environment. The skin plays a relevant role in protection, communication, sensory perception, locomotion, respiration, ion regulation excretion, and thermal regulation [11]. Likewise, the fish skin also plays a vital role in medical applications such as regenerating tissue in trauma, chronic wound-like burns, and diabetic wounds [12].

Recently, the fish skin has been used broadly in the management of skin burns. Nile *Oreochromis niloticus* fish skin which is commonly called tilapia has been used widely in medical applications [13]. This fish is commonly found and cultivated in Thailand and Brazil. This fish skin has the highest collagen content, i.e., collagen types I and III and Omega-3, which helps in the speedy recovery of burn [14, 15]. The Omega-3 content has been found to proliferate keratinocytes that enhance the wound healing process [16]. Collagens are the major proteins of extracellular matrix (ECM) which are the most abundant fibrous proteins in fish. ECM is a three-dimensional network of extracellular macromolecules, such as collagen, enzymes, and glycoproteins, that provide structural and biochemical support of surrounding cells [17]. Similarly, Omega 3 helps in preventing extensive wound. Likewise, keratin fights against disease-causing microbes [18].

Researchers in Brazil created a sterilized tilapia skin wound dressing and carried out clinical trials for second and third-degree burns. So far, more than 56 patients have received this treatment [19]. Recently, tilapia collagen nanofibers were found to quickly and effectively promote the skin wound healing process and were also shown to promote cell adhesion, proliferation, and differentiation in rats [20]. Moreover, another study carried out in vitro and in vivo experiments for wound healing evaluation and the results illustrated the promising application of marine collagen peptides from the tilapia skin [21].

In present days, the biosynthesis methods using plant extract are being immensely utilized in order to synthesize nanoparticles over the common chemical and physical methods [22]. Physical and chemical synthesis that uses speed, radiation, and chemicals as reducing agents requires high temperature, energy, and time to obtain nanoparticles [23]. Moreover, the toxic chemicals used in the chemical synthesis process likely generate harmful waste that might affect internal health if used as biomedical applications [24, 25]. Likewise, chemically synthesized nanoparticles barely show any therapeutic properties [26]. On the other hand, the biosynthesized nanoparticles contain medicinally active phytochemicals from the plants that can be used as therapeutic agents [27, 28]. In addition, plants have a complex network of antioxidant metabolites and enzymes that act together to protect cellular components from oxidative damage. This method is also eco-friendly, cost-effective, and follows an easy procedure comparatively with high yields [29, 30]. Researchers have been exploring different resources available in the environment such as microorganisms, plants and plant extracts, different templates as virus's DNA, membranes, and diatoms to obtain the desired form of nanoparticles as per the field of application.

Plant extracts offer high-quality manipulation and control over crystal growth and their stabilization. In order to obtain nanoparticles with desired shape, size, and dispersity, the biosynthesis methods are performed using plant extracts [31]. Various plants such as *Medicago sativa*, *Aloe vera* leaves, *Azadirachta indica* leaves, *Camellia sinensis*, *Capsicum annuum*, *Cinnamomum camphora* leaves, *Datura metel*, *Emblica officinalis* fruit, *Ocimum sanctum,* and *Geranium* leaves have all been used to synthesize various metal nanoparticles [32, 33]. When the metal core is capped with biological components, the therapeutic activity of these nanoparticles synthesized by plant-mediated synthesis is significantly improved [6].

*Aloe vera* extracts can be found in a wide range of medicinal and dermatological treatments. Many studies show that the *Aloe vera* herb may effectively heal burns, sunburns, inflammatory skin diseases, and wounds when used topically [34]. Monosaccharides and polysaccharides, tannins, sterols, organic acids, enzymes, saponins, vitamins, and minerals are all found in *Aloe vera* [35]. Alpine, an anthraquinone heterosis, is the most active component in *Aloe vera* plant extract. Many researchers mentioned that *Aloe vera* inhibits the growth of some microorganisms, such as *Streptococcus pyogenes* and *Shigella flexneri*, against Gram-positive bacteria that cause food poisoning or diseases in humans and animals [36]. AgNPs synthesized with *Aloe vera* extract helps to incorporate beneficiary components of *Aloe vera* in nanoparticles for the better healing process of burn wound [3].

Many illnesses in humans are associated with the accumulation of free radicals. Antioxidants are substances that supply electrons to damaged cells in order to prevent and stabilize the damage caused by free radicals [37]. It is generally understood that oxidation damages a variety of biological components which creates a variety of illnesses. Thus, several scientific articles have been published which describe the close relationship between the oxidative damage and different illnesses caused by this, such as cancer, liver disease, Alzheimer's disease, aging, arthritis, inflammation, diabetes, Parkinson's disease, atherosclerosis, and AIDS [38]. Hence, antioxidants have been used to treat a variety of illnesses in order to avoid oxidative damage.

The microorganisms are already abundantly found in the environment. These microorganisms can survive under adverse conditions [39]. Antibacterial activity can be determined by the well diffusion method, disc diffusion method, broth dilution method, etc., for *Escherichia coli (E.coli)* and *Pseudomonas aeruginosa (P. aeruginosa)* [40]. AgNPs have antimicrobial activity against *Staphylococcus epidermidis, Pseudomonas fluorescens, Klebsiella pneumonia, Pseudomonas aeruginosa, Salmonella typhi, Proteus vulgaris,* and multidrug resistant bacteria [41]. It also exhibits inhibitory activity against *Bacillus cereus*, *Escherichia hermanii*, *Bacillus subtilis*, agricultural plant pathogens, and antibiotic resistant bacteria [42]. Similarly, different research works confirmed that along with antibacterial properties, biologically synthesized AgNPs also possess antiviral and anticancerous properties [30, 43]. Green synthesized AgNPs of *Phaseolus coccineus L.* and *Micrococcus luteus* have also shown antiviral activity against HSV-1, HAV-10, and CoxB4 viruses. Moreover, biosynthesized AgNPs are effective to fight against lung cancer, breast cancer, prostatic cancer, and many more [44].

To the best of our knowledge, there has not been any report on the composite scaffold incorporating biosynthesized AgNPs on the fish skin for the accelerated burn wound healing process. So, we are motivated to prepare a naturally obtained healing approach by incorporating biosynthesized AgNPs on the fish skin.

## 2. Experiment

### 2.1. Materials

*Aloe vera*, ascorbic acid, calcium chloride (CaCl_2_), deionized water, dextran, distilled water, Dulbecco's Modified Eagle Medium (DMEM), dimethyl sulfoxide (DMSO), 2,2-diphenyl-1-picrylhydrazyl (DPPH), ethylenediaminetetraacetic acid (EDTA), ethanol, foetal bovine serum (FBS), formalin, glutamine, Hank's balanced salt solution (HBBS), liquid nitrogen, methanol, mice fibroblast 3T3 cells, sodium chloride (NaCl), parafilm, phosphate buffer saline (PBS), *P. aeruginosa,* penicillin, raffinose, *S. aureus,* sodium azide, streptomycin, sucrose, tilapia fish, Tris-HCl, Triton X-100, and trypsin.

### 2.2. Biosynthesis of AgNPs

AgNPs were biosynthesized from *Aloe vera* extract using the hydrothermal method.

#### 2.2.1. Preparation of *Aloe vera* Extract

The fresh leaves of an adult *Aloe vera* plant were selected and harvested. The leaf base was cut and left for a few minutes to drain out the yellow resins. Then, the leaves were washed with sterile water and cut into small pieces. After that, 50 g of leaves and 50 ml deionized water were boiled for 20 minutes and cooled at room temperature [3]. The plant extract was filtered using Whatman filter paper and stored in the refrigerator at 4°C. Finally, the obtained plant extract was used as a reducing agent as well as capping/stabilizing agent in the synthesis of AgNPs. The extracted *Aloe vera* which was used for the preparation of AgNPs from the hydrothermal method is shown in Figure S1 (supporting information).

#### 2.2.2. Biosynthesis of AgNPs

The biosynthesized AgNPs were prepared using the hydrothermal method [3]. For this, 50 mg silver nitrate (AgNO_3_) was added in 20 ml deionized water. It was then mixed with 20 ml of extract solution by vigorously stirring at room temperature for 30 minutes. The mixture was then added to a sealed Teflon-lined vessel and incubated at 100°C for 6 hours and 12 hours. A grey precipitate was collected and purified by redispersing with distilled water followed by centrifugation at 12,000 rpm for 15 minutes to obtain a pellet of AgNPs. The centrifuging and redispersing processes were repeated for three times. Finally, AgNPs were obtained upon drying. The reduction of clear AgNO_3_ to brown color solution by *Aloe vera* extract incubated at 100°C for 6 h and 12 h and the obtained corresponding powder of AgNPs are shown in Figure S2 (supporting information).

### 2.3. Characterization of AgNPs

FT-IR analysis was used to analyze the presence of different functional groups of synthesized AgNPs. This is the preferred method of infrared spectroscopy. In this instrument, the monochromator and the slits are replaced by an interferometer, usually of the Michelson type. The different chemical structures produce different spectral fingerprints. Therefore, infrared spectroscopy is one of the most common and widely used spectroscopic techniques which can be used as a fingerprint for the purpose of comparing molecules.

XRD measurement of AgNPs synthesized by the *Aloe vera* leaf was carried out using Cu-K*α* radiation source in the 2*θ* range of 20–80 at room temperature. The primary use of this technique is the identification and characterization of compounds based on their diffraction pattern. XRD relies on the dual wave/particle nature of X-rays to obtain information about the structure of crystalline materials. Thus, this was used to determine the phase variety and grain size of synthesized AgNPs. The crystallite size of the synthesized AgNPs was determined by using Scherrer's equation as follows [45]:

(1) $$\begin{array}{c} D=\frac{K\lambda }{\beta \;\cos\;\theta }\text{,} \end{array}$$

where *D* = average crystallite size, *ß* = line broadening in radians, *λ* = wavelength of X-ray, *θ* = Braggs angle, and *k* = constant.

The prepared AgNPs were further analyzed by UV-Vis spectroscopy for structural characterization. UV-Vis spectroscopic measurements were performed at room temperature using the UV-Vis spectrometer.

#### 2.3.1. Antioxidant Assay

The antioxidant activity of the biosynthesized AgNPs was determined by the free radical scavenging assay, where the free radical used was 2, 2-diphenyl-1-picrylhydrazyl (DPPH). The free radical scavenging activity (RSA) was determined by monitoring the change in optical density (OD) of DPPH radical. For this assay, a stock solution of AgNPs, *Aloe vera* extract, and ascorbic acid was prepared using methanol. Then, a serial dilution was carried out to obtain solutions of 200, 100, 50, and 25 *μ*g/mL. A fresh DPPH solution of 0.2 mM was prepared by dissolving 7.88 mg of DPPH powder in 100 ml of methanol. Then, a volume of 50 *μ*l of each sample (25–200 *μ*g/mL) was mixed with 150 *μ*l of DPPH in a 96-well plate in triplicate. The control was prepared by replacing the same volume of sample with absolute methanol. After 30 minutes of incubation at room temperature, the absorbance was measured at 517 nm using a Chromate ELISA reader. The %RSA was calculated by using the following formula:

(2) $$\begin{array}{c} \%RSA=\frac{control\;absorbance-sample\;absorbance}{control\;absorbance}\times 100\%\text{.} \end{array}$$

### 2.4. Antibacterial Tests

The antibacterial activity was observed on *Pseudomonas aeruginosa* and *Staphylococcus aureus* by the agar well diffusion test and broth dilution method. The AgNPs were used as the test sample, while *Aloe vera* extract and distilled water were used as negative control, and Penicillin was used as positive control. The susceptibility test was evaluated by the agar well diffusion method on agar plates. The agar plates were inoculated with bacterial strains under aseptic conditions, and the punched wells (6 mm diameter) were filled with test samples and incubated at 37°C for 24 hours. After incubation period, the diameter of the growth inhibition zones was measured. The experiments were carried out in triplicates.

The MIC of the AgNPs was checked using 6 test tubes containing bacteria (*S. aureus* and *P. aeruginosa*) grown in a nutrient broth. The 2 ml of bacteria containing broth was taken in all the tubes. The MIC of AgNPs was tested through the serial twofold dilution's method. The colloidal AgNPs solution with the concentrations from 12.5 *μ*g/ml to 200 *μ*g/ml were taken (i.e., 12.5 *μ*g/ml, 25 *μ*g/ml, 50 *μ*g/ml, 100 *μ*g/ml, and 200 *μ*g/ml) and added at the test tubes and incubated for 8–10 hours. The MIC was determined by the evaluation of turbidity of tubes with a constantly increasing concentration of antimicrobial agents. The point at which no turbidity was observed was taken as MIC. Then, MBC was measured after MIC determination. For this, AgNPs were pipetted onto nutrient agar plates and incubated at 37°C for 24 hours. The MBC value was interpreted at the lowest concentration of colloidal AgNPs at which inoculated bacterial strains were completely killed.

### 2.5. Preparation of Decellularized Fish Skin Scaffold

Tilapia fish were obtained from the Centre for Aquaculture-Agriculture Research and Production Pvt. Ltd (CAARP), Chitwan, Nepal. The adult tilapia fish, extraction skin tissue from tilapia fish, and decellularized fish skin scaffold are shown in Figure S3 (supporting information). At first, the fish was descaled and the skin was peeled with the help of a sharp knife. Then, the skin samples were washed two times with sterile phosphate buffer saline (PBS) containing 50 mm ascorbic acid and 500 ppm streptomycin to make it germ-free. Thus, the prepared skin sample was used for further processing [46].

For decellularization, the skin sample was incubated in a PBS solution containing 0.5% Triton X and 0.02% sodium azide for 2 hours in 40 rpm at room temperature. Then, the sample was washed two times with Hank's balanced salt solution (HBSS) for 10 minutes in 40 rpm at room temperature. The sample was again treated with a decellularizing solution containing 0.5% sodium dodecyl sulphate (SDS) for 1 hour at the same rpm and temperature. The resultant sample was washed with PBS [46].

Then, the decellularized skin sample was incubated in a digestion containing 1 M Tris-HCl and 0.05 g/ml trypsin maintained at a pH of 8.5 for 2 hours at room temperature. Then, the resultant decellularized skin sample was immersed in a prefreezing solution containing 7% dextran, 6% sucrose, 6% raffinose, and 1 mm EDTA in HBSS and stored at −20°C. Finally, these prepared samples were cut into different dimensions as required before being used for testing.

#### 2.5.1. Histological Analysis

Histological analysis was carried out to ensure the decellularization of fish skin scaffold. The skin specimen was embedded in paraffin blocks and sectioned with microtome. Sections of the fish skin and decellularized fish skin scaffold were mounted on glass slides separately and stained with hematoxylin and eosin (H and E).

The slides that were fixed using formaldehyde were dipped in distilled water. They were then transferred to the beaker containing haematoxyl in solution for 10 minutes. Those were then transferred to the beaker under running tap water for 10 minutes. They were dipped in the acid alcohol (1 ml of HCl in 90 ml of 70% ethanol) unless they were converted to red. The slides were then kept under the running tap water for 30 minutes. Thus, obtained slides were transferred to the beaker containing 80 ml of distilled water and were left for 10 minutes. Then, they were dipped into a beaker containing eosin for 1 minute and were transferred to the beaker containing distilled water (80 ml) where those were only dipped. The slides were then dipped in 70% ethanol followed by 95% and then absolute alcohol. Thus, resultant slides were then air-dried and dipped in the beaker containing xylene I for 10 minutes. Again, it was transferred to the beaker containing xylene II for 10 minutes. Finally, a drop of DPX was added on the top of the resultant slide and was covered gently with the cover slip so that there is no formation of bubbles.

### 2.6. In Vitro Degradation Rate Test

The DFS scaffolds were cut into 2 cm × 2 cm dimensions. The dry weights of the scaffolds were taken. Then, the scaffolds were kept in 2 ml PBS solution (pH 7.4) under aseptic conditions in a sealed tube and incubated at 37°C for 1, 2, 3, 4, 5, 6, 7, 14, 21, and 28 days. After each time point, scaffolds were taken out and dried and weighted.

The degree of degradation of each scaffold was defined as the weight loss percentage calculated by the following equation:

(3) $$\begin{array}{c} Weight\;Loss\;\%=\frac{W_{O}-W_{t}}{W_{O}}\times 100\%\text{,} \end{array}$$

where *W*_0_ denotes initial weight of the dried scaffold and *W*_*t*_ denotes the weight of degraded scaffold after respective time intervals.

### 2.7. Swelling Test

For the swelling test, the dry weights of the scaffolds were recorded before immersion. Then, the scaffolds were immersed in PBS solution (pH 7.4) under aseptic conditions in a sealed tube and incubated at 37°C for 24 hours. Then, the wet weights of the scaffold were recorded. The degree of the swelling capacity was calculated using the following equation:

(4) $$\begin{array}{c} Swelling\;ratio=\frac{W_{O}-W_{t}}{W_{O}}\text{,} \end{array}$$

where *W*_0_ represents the initial weight of the dried scaffold before immersion and *W*_*t*_ represents the weight of the scaffold after immersion.

### 2.8. Moisture Content Test

The DFS scaffolds were cut into 2 cm × 2 cm dimension. The initial weights of the scaffolds were recorded. Then, the samples were kept in a desiccator containing calcium chloride (CaCl_2_) until they reached the constant dry weight. The amount of moisture contained in the scaffold was defined as the weight loss of scaffold upon drying in the desiccator.

### 2.9. Mechanical Test

A folding endurance test was performed to test the mechanical strength of the DFS scaffold. Six DFS scaffolds were subjected to repeat folding at the same place until it breaks. The folding endurance was given by the number of times the sample could be folded at the same place without breaking.

### 2.10. Permeability Test

An apparatus was created by using two test tubes. The scaffold of 2 cm × 2 cm was wrapped between the test tubes. One test tube was filled with saltwater for higher concentration, and another was filled with distilled water for lower concentration. After 24 hours, levels of both the liquids were observed. Permeability of scaffold was calculated by measuring the increased volume of salt solution. The experiment was carried out in triplicates.

### 2.11. Doping of AgNPs in Decellularized Fish Skin Scaffold

2 mg of AgNPs was prepared in 2 ml distilled water. Uniformly dispersed colloidal AgNPs were prepared by sonicating in a bath sonicator for 30 minutes at 30°C. Using the double dilution method, four concentrations of colloidal AgNPs were prepared (50 *μ*g/ml, 100 *μ*g/ml, 150 *μ*g/ml, and 200 *μ*g/ml).

The DFS scaffolds were cut into different dimensions before doping with AgNPs. The DFS scaffolds were then soaked in all of the concentration of the uniformly dispersed colloidal AgNPs solution for 24 hours at 50 rpm in room temperature. The prepared scaffolds were used for *in vitro* testing.

#### 2.11.1. AgNPs' Loading Capacity Test of DFS Scaffold

To find out the amount of colloidal AgNPs loaded in the prepared DFS scaffold, 200 *μ*l of different concentrations of each AgNP solution were kept in a 96-well plate and absorbance was recorded at 450 nm using Chromate ELISA reader. Similarly, 200 *μ*l of each AgNP solution after doping was kept in a 96-well plate and absorbance was recorded. The experiments were carried out in triplicates.

The amount of colloidal AgNPs absorbed into the DFS scaffold was determined by comparing the absorbance of the AgNPs solution before and after doping. The AgNPs' loading capacity of DFS scaffold was calculated as the amount of the absorbed colloidal AgNPs per square millimeter of DFS scaffold (*μ*g/mm^2^).

#### 2.11.2. AgNPs' Release Rate Profile

DFS scaffolds doped with AgNPs were placed in 2 ml PBS solution and incubated at 37℃, and the absorbance values of PBS solution at 2, 4, and 24 hours were recorded at 450 nm Chromate ELISA reader. The changes in the absorbance were observed to determine the rate of AgNPs released from the scaffold. The experiments were carried out in triplicates, and a sample without AgNPs was used as a control.

### 2.12. In Vitro Testing

For the in-vitro testing, a cytocompatibility test must be performed. The biocompatibility of biomaterials is mainly determined by its cytotoxicity and hemocompatibility. The prepared samples should not cause any toxic reaction and immunological rejection in the body so that the biocompatibility test should be needed for those samples. Therefore, the individual materials of the fish skin scaffold and the nanoparticles doped fish skin scaffold should need the biocompatible test to ensure effective and safety uses for humans. This includes the cytotoxicity test, sensitization assay, hemocompatibility test, implantation test, irritation test, acute systemic toxicity, subchronic toxicity, genotoxicity, carcinogenesis bioassay, reproductive and developmental toxicity, pharmacokinetics, and preclinical safety test.

#### 2.12.1. Cytotoxicity

Cytotoxicity assays are utilized during drug development before toxicological testing is performed. These assays are additionally used for controlling the quality of manufactured drug compounds. There are quantitative and qualitative methods of cytotoxicity testing. The quantitative cytotoxicity assay mainly used is the MTT assay, and the qualitative assays used are the MEM elution method, the direct contact method, and the agar diffusion method.

#### 2.12.2. MTT Assay

The cytotoxicity assay uses 3-(4, 5-dimethylthiazol-2-yl)-2, 5-diphenyltetrazolium bromide dye, commonly referred to as MTT. MTT is a yellow-colored water-soluble compound which is split by mitochondrial succinate dehydrogenase, giving rise to the violet-colored formazan [47]. The conversion of MTT to formazan only occurs in viable cells. The formazans are insoluble in water but soluble in solvents such as dimethyl sulfoxide (DMSO) and isopropanol. After formazans are dissolved, the solution is taken for spectrophotometry. The solution is kept in cuvette and kept in the spectrophotometer to find absorbance. The absorbance of the solution is directly proportional to the concentration of the solution. If the solute in the solution is high, there will be a greater number of viable cells, whereas the lesser concentration, the number of viable cells will be less.

Here, the prepared scaffold was placed in 3 different 96-well plates. Fibroblast 3T3 cells were seeded in each well plate. The plate was then incubated for 48 hours at 37°C, and 100 of 5 mg/ml MTT was added in the well plate. The plate was again incubated for 4 hours, and 15% DMSO was added to it. The absorbance reading in 96-well plates was carried out in chromate ELISA Reader supplemented with Chrome Manager Software at 595 nm.

## 3. Results and Discussion

### 3.1. FT-IR Analysis

**Figure 1:**
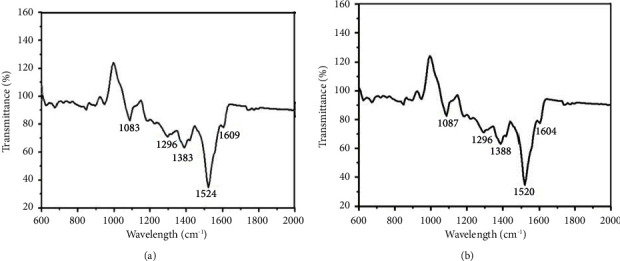
FT-IR spectra of biosynthesis AgNPs using *Aloe vera* leaf extract in 6 hours (a) and 12 hours (b) at 100°C.

The FT-IR analysis of AgNPs synthesized in 6 hours and 12 hours at 100°C is shown in Figure 1. As shown in the figure, both graphs have similar absorbance bands within the same range. Here, strong absorbance bands were observed at 1609 cm^−1^,1524 cm^−1^, 1383 cm^−1^, and 1083 cm^−1^ which correspond to nitro compound, alkane, and amine, respectively [48]. The band at 1609 cm^−1^ arises due to C-N and C-C stretching indicating the presence of proteins [49]. The band around 1524 cm^−1^ and 1383 cm^−1^ corresponds to C=O stretching vibration (Amide I) and N-H bending vibrations (Amide II), and the band around 1296 cm^−1^ corresponds to the CH_2_ wagging vibrations (Amide III) [50]. 1083 cm^−1^ corresponds to -C-O- of the ester group [51]. The peaks observed in the range of 1000–450 cm^−1^ confirm the CH group [50].

Thus, the presence of biological molecules such as proteins confirmed the bio-fabrication of AgNPs. It also suggests that the proteins are responsible for the capping and stabilization of the synthesized AgNPs [52].

The other medium to small peaks shows the presence of a halo compound and alcohol. This in total shows that *Aloe vera* successfully reduced AgNO_3_ into AgNPs as well as capped the nanoparticle as most of the compound shown on the surface of the nanoparticle of *Aloe vera*.

### 3.2. XRD Analysis

**Figure 2:**
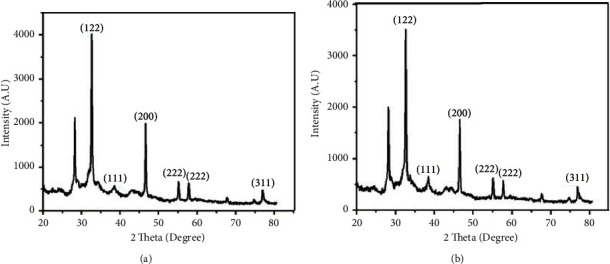
XRD patterns on AgNPs synthesized using *Aloe vera* leaf extract solution. (a) 6 hours hydrothermal at 100°C; (b) 12 hours hydrothermal at 100°C.

The XRD patterns of AgNPs are prepared by using the two hydrothermal conditions, i.e., 100°C for 6 hours and 12 hours as shown in Figures 2(a) and 2(b), respectively. The XRD analysis of synthesized AgNP extract from the *Aloe vera* leaf shows different peaks ranging from 30^0^ to 80°. The peaks are indexed with reference to the standard JCPDS card no. 04-783 for silver that are found to be 122, 111, 200, 222, and 311 around 32.5°, 38.3°, 50°, 60°, and 80°, respectively, corresponding to the cubic face of AgNPs [52, 53]. The peaks observed around 27° and 32° may be due to the leaf extract. These Braggs peaks might have resulted due to the capping agent, stabilizing the nanoparticles [54]. The previous research works have showed that the highest peaks of green synthesis AgNPs were found between the ranges of 30‒40 radians [52, 55]. The crystallite sizes of the AgNP particles prepared from 100°C for 6 hours and 12 hours are calculated using the Debye–Scherrer's equation which were found to be 29.1 nm and 35.2 nm, respectively [56]. The XRD analysis data of the peak position, full-width-half-maximum (FWHM), size, and average size of both samples of AgNPs are shown in Table S1 (supporting file).

### 3.3. UV-Vis Spectroscopy

**Figure 3:**
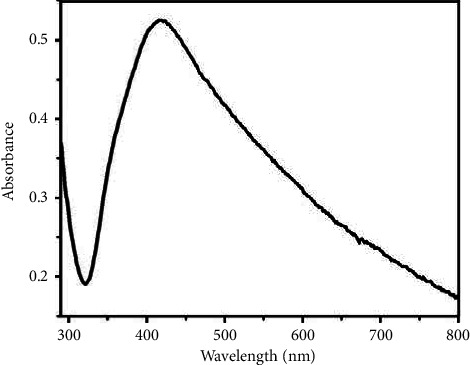
UV-Vis spectra of AgNPs.

The surface plasmon spectrum of synthesized AgNPs is shown in Figure 3. The figure illustrates that the AgNPs displayed a strong characteristic surface plasmon resonance band in the visible region, centered from 417 to 424 nm which is specific for AgNPs. Similarly, the electronic transition of metallic silver appears in the range of around 300‒330 nm. Besides these two peaks, there are not any other peaks, which indicate the absence of nanoparticle aggregation. Thus, UV-Vis spectroscopy further confirms the formation of silver nanoparticles [57, 58].

### 3.4. Antioxidant Assay

Free radical scavenging activity of the AgNPs was assessed by the DPPH assay. The freshly prepared DPPH solution exhibited a deep purple color with maximum absorbance at 517 nm. The disappearance of purple color on adding synthesized AgNPs might be due to the presence of antioxidants in the medium. The different color during the antioxidant assay of biosynthesized AgNPs and *Aloe vera* extract compared with chemically synthesized AgNPs and ascorbic acid is shown in Figure S4 (supporting information).

**Figure 4:**
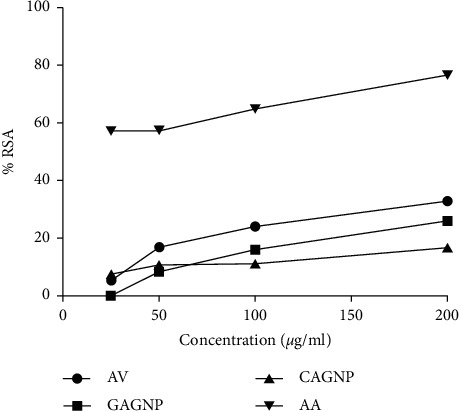
Free-radical scavenging activity of *Aloe vera* extract, AgNPs using *Aloe vera* leaf extract (GAgNPs), chemically synthesized AgNPs (CAgNPs), and ascorbic acid (AA). The ability of *Aloe vera* extract, GAgNPs, CAgNPs, and AA (used as the reference antioxidant) to inhibit DPPH was estimated as a function of increasing concentration (from 50 to 200 *μ*g/ml) and by recording the decrease in absorbance at 517 nm.

Here, Figure 4 illustrates that the %RSA values are different between the values of *Aloe vera* extract and CAgNPs and between the values of GAgNPs and CAgNPs. Free radical scavenging activity of *Aloe vera* extract, AgNPs prepared from *Aloe vera* extract, and chemically prepared AgNPs on DPPH radical was found to increase in concentration, showing a maximum of 33%, 26%, and 17%, respectively, at 200 *μ*g/ml. The standard ascorbic acid, however, at this concentration exhibited 76% inhibition, but it cannot be used in a burn wound treatment. The IC50 value of *Aloe vera* extract, AgNPs prepared from *Aloe vera* extract, and chemically prepared *Aloe vera* extract was found to be 307 *μ*g/ml, 362 *μ*g/ml, and 620 *μ*g/ml, respectively, whereas the IC50 value of ascorbic acid was 7 *μ*g/ml. The experimentally obtained data of DPPH radical scavenging activity (%RSA) and IC50 value of *Aloe vera* extract, biosynthesized silver nanoparticles using *Aloe vera* leaf extract, chemically synthesized silver nanoparticles, and ascorbic acid (standard) are shown in Table S2 (supporting information). These obtained results are comparable with a scientific article published by Barabadi et al., where the %RSA of green synthesized AgNPs from *Cestrum nocturnum* was found to be 29.5% [43].

The previous result also demonstrates that the scavenging activity of *Aloe vera* extract is higher than GAgNPs and CAgNPs which might be due to the presence of antioxidant phenolic compounds, flavonoids, ascorbic acid, *β*-carotene, and *α*-tocopherol in the *Aloe vera* extract [59]. In addition, the GAgNPs have greater scavenging activity than that of CAgNPs, further confirming that the antioxidant property of *Aloe vera* extract has made an important contribution in the increment of scavenging activity of AgNPs.

Burn wound on the skin is reported to be an oxidation process which generates free radicals from various cellular pathways [60]. Thus, the antioxidant GAgNPs would help in eradicating the free radicals from the wound and provide a healthy environment for the skin to heal.

### 3.5. Histological Analysis

**Figure 5:**
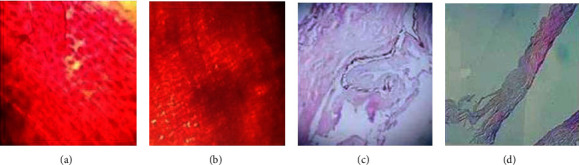
H&E staining of the fish skin before and after decellularization. Cross-sectional analysis of the fish skin at 10x: (a) before decellularization and (b) after decellularization. The sagittal section analysis of the fish skin at 40x: (c) before decellularization and (d) after decellularization.

Figure 5 illustrates the digital image of the histological slide of the fish skin before and after decellularization. The histological study of slides under the microscope shows that the decellularization of the fish skin with different decellularizing chemicals such as Triton-X, SDS retained the ECM components without any evidence of cellular and nuclear materials. Likewise, the H&E stain results of fish skin tissue before and after decellularization confirmed the suitable maintenance of the ECM structure in DFS scaffold. Moreover, the decellularization procedure removed the cells from the tissue and created more porous scaffolds as shown in Figure 5. This facilitates the absorption of metal nanoparticles for the bioscaffold development and application. The porous structure of the scaffold provides the large surface area for the cell attachment.

The decellularization procedure removes the cells as well as ensures the scaffold is free from nuclear content as well as microbial contaminations. The complete decellularization of the scaffold was ensured by histological analysis of the scaffold using the H&E staining technique. The absence of nuclear content and the presence of an intact ECM structure only are confirmed by the absence of purple stain and the presence of pink stain. The histological slide of the fish skin sample showed the purple stain that indicates the presence of nucleus, whereas the histological slides of the DFS scaffold showed the lesser presence of purple stain than pink stain, which indicates the decrease in nucleus content. The DFS scaffold showed a mesh-like-reticular arrangement of fibers. The absence of cells in the ECM matrix results in porosity which facilitates adsorption of AgNPs and provides a large surface area for cell attachment for better proliferation.

### 3.6. Contamination Test

**Figure 6:**
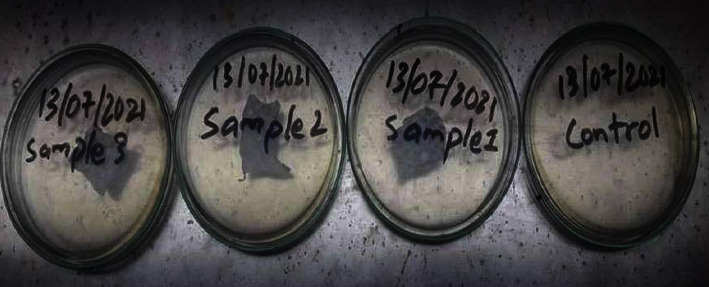
The contamination test of DFS scaffold. The test was performed on three identical DFS scaffolds, and there was no bacterial growth on the sample.

Figure 6 shows the result of the contamination test. There were not any colonies of bacteria observed in the media even after 24 hours of incubation at 37°C which is an appropriate condition for bacterial colony formation. This proved that the DFS scaffolds were free from microbial contaminations and can be safely used for in vitro experiments.

### 3.7. In Vitro Degradation Rate Analysis

**Figure 7:**
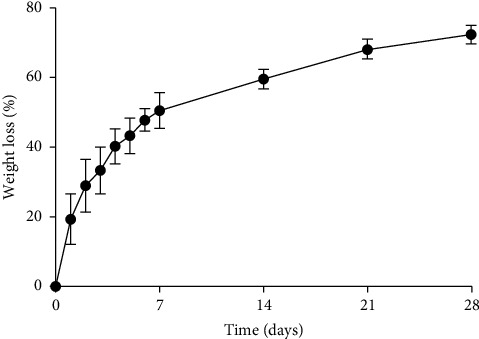
Percentage weight loss of the DFS scaffold during in vitro degradation at 37°C in PBS solution maintained at pH 7.4. The degradation rate was measured as weight lost by the scaffold per day.

The graph was plotted according to the data obtained from the experiment which is shown in the Figure 7. The degradation rate of the DFS scaffold was 19.4%, 29%, 33%, 40.3%, 43.4%, 47.9%, and 50% in the first successive 7 days, respectively. The degradation rate of DFS scaffold was increased constantly by about 9% within 2^nd^ and 3^rd^ weeks. After the 3^rd^ week, slow degradation was perceived and about 70% of the scaffold was degraded up to 28 days. The experimental data for percentage weight loss of the DFS scaffold during in vitro degradation at 37°C in PBS solution with a pH of 7.4 up to the 3^rd^ week are shown in Table S3 (supporting information).

A degradation test is used to know the stability of the scaffold during contact with the wound. An ideal scaffold is supposed to degrade at a rate proportional to the healing rate of the wound. Burn wounds are usually healed within 14 to 21 days. If the scaffold lasts up to 21 days, then it can be used as a treatment for burn wounds [61].

The DFS scaffold is ECM based scaffold that consists of almost 80% collagen. The degradation of the DFS scaffold was similar to that of the collagen-based scaffold [62]. The error bars show sample standard deviation from triplicate measurements.

### 3.8. Swelling Analysis/Water Uptake Analysis

**Figure 8:**
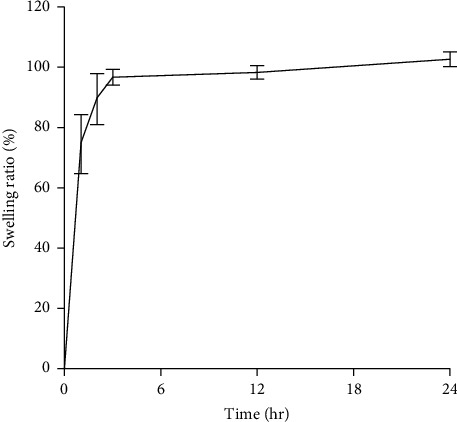
Swelling ratio or water uptake capacity of DFS scaffold. The water uptake capacity was determined by the amount of PBS solution absorbed by the scaffold per hour in a pH and temperature-maintained condition. Error bars are the standard deviation from triplicate measurements.

Figure 8 shows the swelling ratio graph from the obtained results, and the corresponding data are shown in Table S4 (supporting information). Also, the dried DFS scaffold in desiccators after the swelling test is shown in Figure S5 (supporting information). The DFS scaffold showed a mean swelling capacity of 74.7%, 89.6%, 96.86%, 98.45%, and 102.89% in 1, 2, 3, 12, and 24 hours, respectively. An ideal scaffold is required to have a water absorption/water uptake capacity of 100‒800% to prevent the buildup of a fluid and enhance the formation of new ECM [61].

Hydrophilicity and the microstructure of a scaffold are the key determinants of a scaffold's water uptake capacity [63]. Since the prepared DFS scaffold contains an enormous amount of collagen fiber, the scaffold was easily wettable by polar solvents such as PBS. Collagen contains a large number of functional groups which are capable of binding water; therefore, they exhibit a high swelling ability. Likewise, the porous microstructure of collagen fiber facilitates water uptake, making the scaffold supportive for the wound healing process [62]. The PBS solution having a pH of 7.4 that corresponds to the body's internal pH (e.g., blood) supports to examine the behavior of the material inside the body. The high swelling capacity of prepared DFS scaffold verified that it has a significant ability to uptake the excessive wound exudates. The obtained result suggests that the DFS scaffold possesses porous lamellar matrix spaces which increased the water containing capacity. Thus, the porous structure creates a suitable ambience for cell proliferation when used to heal the burn wound.

### 3.9. Moisture Content

**Figure 9:**
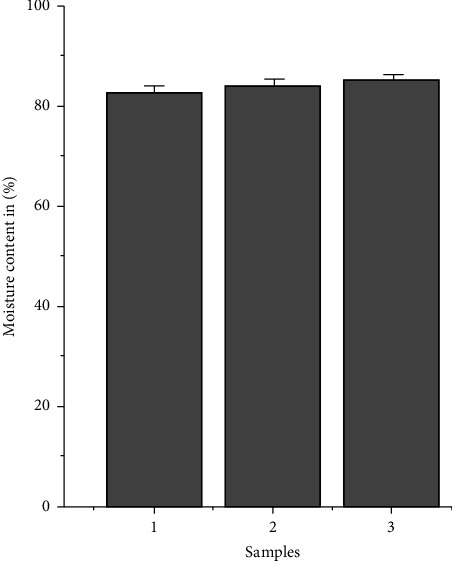
Moisture content of DFS scaffold. The moisture content of DFS scaffold was found to be 81.7 ± 3.6%. The graph is presented as the amount of moisture in % present per identical sample (*N* = 3). The result is expressed as grams of water in 100 g of dry sample weight. Error bars are the standard deviation from triplicate measurements.

Figure 9 illustrates the moisture content of different samples. According to the graph shown in figure, the DFS scaffold possessed a high moisture content of 81.7 ± 3.6%. The experiment was performed in triplets, and the result is presented as the mean ± standard deviation. The data of initial and final weight of DFS scaffold required to calculate moisture content percentage are given in Table S5 (supporting information).

The appropriate moisture content in the DFS scaffold ensures the sufficient supply of moisture to the wound. A moist environment has been proven to enhance the wound healing process by promoting angiogenesis, cell adhesion, growth, migration, and collagen synthesis and facilitating re-epithelization and cessation of dead tissue and fibrin [64].

### 3.10. Permeability

**Figure 10:**
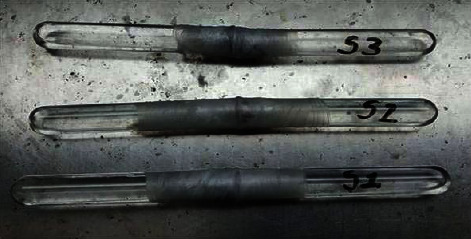
The permeability test of the DFS scaffold of different samples.

The experimental setup of the permeability test is shown in Figure 10. It was observed that there was no increment of volume of liquid in all three samples. The permeability test of the DFS scaffold showed that the scaffold is impermeable to the water and external environment. Correspondingly, the loaded AgNPs prevent infection and decrease bacterial load by their intrinsic antibacterial properties. This ensures that the scaffold is the perfect barrier for the protection of burn wounds against external microbial contamination.

### 3.11. Mechanical Test

The mechanical test of the DFS scaffold showed that the prepared scaffold has high tensile strength, is highly flexible, and is easy to handle by clinicians. The high tensile strength of the scaffold means the mechanical integrity of the scaffold is preserved to a proper level even after the chemical decellularization process. The folding endurance tests demonstrate that the scaffold does not break even after the repeated folding of the scaffold more than 1500 times.

### 3.12. AgNPs' Loading Capacity of DFS Scaffold

The doping of AgNPs on the DFS scaffold depends upon the concentration of a solution of AgNPs and the area of the DFS scaffold. The doping of AgNPs adds antibacterial properties to the DFS scaffold and improves the inflammatory response during the initial phase of treatment. The controlled release of AgNPs from the DFS scaffold is essential to prevent infection due to opportunistic bacteria and guide the wound healing process. The smallest size of AgNPs obtained from incubating 6 h was used to load in the fish skin scaffold.

**Figure 11:**
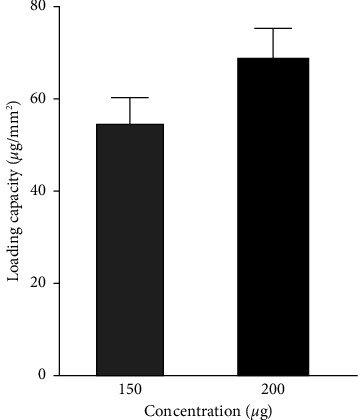
Determination of AgNPs' loading capacity on DFS scaffold at different concentrations. Error bars are the standard deviation from triplicate measurements.

The loading of AgNPs on DFS at different concentrations is shown in Figure 11. The figure illustrates that the loading of AgNPs in 24 hours was 48.7 *μ*g/mm^2^ and 61.5 *μ*g/mm^2^ when doped in 150 *μ*g/ml and 200 *μ*g/ml, respectively. The required data for calculation of AgNPs' loading capacity on DFS Scaffold are shown in Table S6 (supporting information). The results showed that the loading amount of AgNPs depends on the concentration of nanoparticles in the solution in which DFS scaffold is dipped. The porous structure of the DFS scaffold might have facilitated the loading amount of nanoparticles in the scaffold.

### 3.13. AgNPs' Release Rate Profile

**Figure 12:**
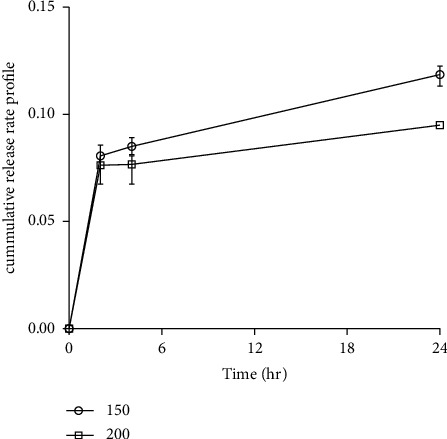
The AgNPs' release profile from DFS scaffold at different time intervals. Error bars shown in figures are the standard deviation of triplicate measurements.

Figure 12 demonstrates the absorbance taken at different time intervals of the prepared scaffold. The release rates of the scaffold in 2, 4, and 24 hours were observed in a significant amount through the absorbance of the fluid at 430 nm which is shown in Figure 12. The experimental data of the AgNPs' release profile from DFS scaffold at different time intervals are shown in Table S7 (supporting information).

The release rate of DFS scaffold doped in 150 *μ*g AgNPs is greater than that of DFS scaffold doped in 200 *μ*g AgNPs over the time period of 24 hours. Specifically, most of the AgNPs were released in a burst in the first 2 hours, with approx. 45 and 50.5% of the AgNPs being released from the DFS scaffold. After the burst release phase, slow release was sustained for 24 hours. One potential reason for this behavior is that the collagens present on the DFS may serve as a steric barrier that retards the diffusion of AgNPs from the scaffolds [65]. Another potential reason for this behavior might be due to the stability of the biosynthesized AgNPs. Alternatively, the sustained release of AgNPs may result from the slow degradation of the collagen scaffolds.

### 3.14. Antibacterial Assay

**Figure 13:**
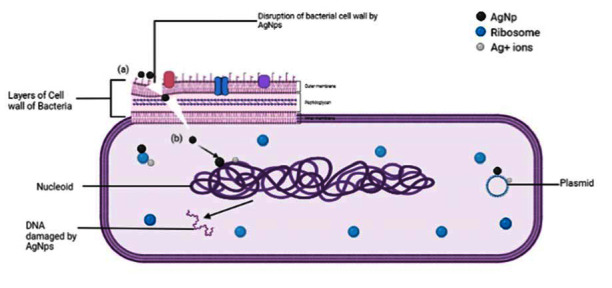
The schematic diagram of the antibacterial activity of green synthesized AgNPs.

*S. aureus*, *P. aeruginosa*, *E.coli*, *Enterobacter*, *A. baumannii,* and *K. pneumonia* are reported to be the most prevalent bacterium in a burn wound [66]. Among these, the Gram-negative bacterium, *S. aureus,* is found to be more active during the wound healing process. So, antibacterial tests were performed in *P. aeruginosa*, Gram-negative bacteria and *S. aureus,* and Gram-positive bacteria. For this, the AgNPs incubated for 6 h were used which was the smallest sized nanoparticle among the samples. The antibacterial effect of AgNPs is influenced by various factors such as shape, size, and colloidal state and surface charge. Reports suggest that the smaller the size of nanoparticles (<30 nm), more effective will be the antibacterial property [67]. For example, the biosynthesized AgNPs from *Zataria multiflora* with an average size of 25.5 nm showed a MIC of 4 *μ*g/ml against *S. aureus* that was lower than the commercial AgNPs [68]. Similarly, the biosynthesized AgNPs which reduced from polyphenol-rich plant extract, with an average size of 8.5 nm, exhibited the MIC of 1.25 *μ*g/ml against *P. aeruginosa*. Likewise, photosynthesized nanosized Ag particles with an average diameter of 10–30 nm showed great antibacterial activity against *A. baumannii* with MIC and MBC of 62.5 and 250 *μ*g/mL, respectively [69, 70]. Meanwhile, among different shapes of nanoparticles, spherical nanoparticles were found to be more effective against bacteria. Smaller and spherical nanoparticles tend to interact with the bacterial cell wall, damage the lipid bilayer, and enter inside the cell resulting in cell death [71, 72]. A study found that nanoparticles show higher antibacterial activity in colloidal form than in noncolloidal form. In a comparative study between cationic, anionic, neutral, and uncoated AgNPs, the cationic AgNPs showed the strongest antibacterial activity. This suggests that the surface charge also affects the activity of nanoparticles [73].  Figure 13 shows the schematic illustration to represent the proposed antibacterial mechanisms of silver nanoparticles.

In the previous figure, AgNPs are attached to the bacterial cell wall, and we constantly infiltrate it (a). This causes physical damage to the cell wall leading to leakage in the cellular content that leads to bacterial death. AgNPs penetrate inside the cell and interact with biomolecules such as ribosomes, nucleoid, plasmids, etc., (b), which causes an increase in reactive oxygen species (ROS). AgNPs also release Ag^+^ ions, which also interact with biomolecules of the bacterial cell. Increased ROS leads to DNA damage, apoptosis, reduction in ATP generation, and lipid peroxidation, thus leading to bacterial death [74].

#### 3.14.1. Susceptibility Test

**Figure 14:**
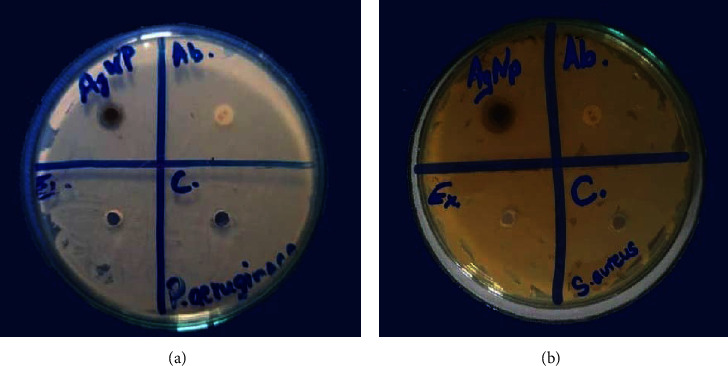
The antibacterial activity assay of biosynthesized AgNPs against (a) *P. aeruginosa* and (b) *S. aureus*. AgNPs showed antibacterial activity against both bacteria. Here, AgNPs, Ab, Ex, and C stand for silver nanoparticles, antibiotic, *Aloe vera* extract, and control, respectively.

The disk diffusion susceptibility test on ager petri plates exhibited a significant zone of inhibition with AgNPs solution which shown in Figure 14. The mean diameter of the zone of inhibition was measured in millimeters (mm) and found to be 17.66 ± 1.5 mm for *S. aureus* and 8 ± 1 mm for *P. aeruginosa,* while penicillin (standard antibiotic) and *Aloe vera* extract did not show any antibacterial activity. The experiment was performed in triplets, and the result is presented as mean ± standard deviation. Thus, this showed that the prepared AgNPs possessed antibacterial properties (Figure 14).

#### 3.14.2. MIC

**Figure 15:**
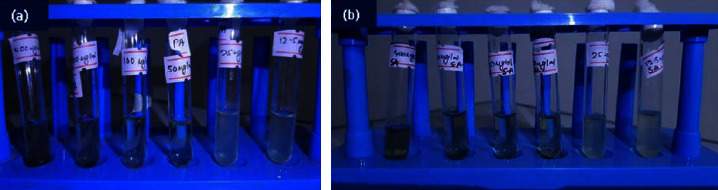
The broth dilution method for the determination of MIC of AgNPs on (a) *P. aeruginosa* and (b) *S. aureus*. The test tube from right to left contains 12.5 *μ*g/ml, 25 *μ*g/ml, 50 *μ*g/ml, 100 *μ*g/ml, 200 *μ*g/ml, and 400 *μ*g/ml of AgNPs, respectively. Turbidity was seen from 50 *μ*g/ml concentration in both bacteria.

The MIC of AgNPs was examined in *P. aeruginosa* and *S. aureus* bacteria by using the broth dilution method seen in Figure 15. The MIC of AgNPs was indicated by the occurrence of a clear solution in the test tube. The turbidity was seen from 50 *μ*g/ml AgNPs in both bacteria. Thus, we can conclude that the MIC of AgNPs is 50 *μ*g/ml.

#### 3.14.3. MBC

**Figure 16:**
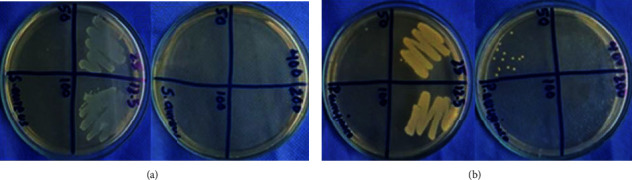
Determination of MBC of AgNPs against *S. aureus* (a) and *P. aeruginosa* (b). The MBC of AgNPs against both was found to be 100 *μ*g/ml.

The MBC result of AgNPs against *S. aureus* and *P. aeruginosa* showed no bacterial growth upto 50 *μ*g/ml of AgNPs which is shown in Figures 16(a) and 16(b), respectively. From this experiment, we can conclude that biosynthesized AgNPs have bactericidal effects as well.

### 3.15. MTT Assay/Cell Proliferation Assay

**Figure 17:**
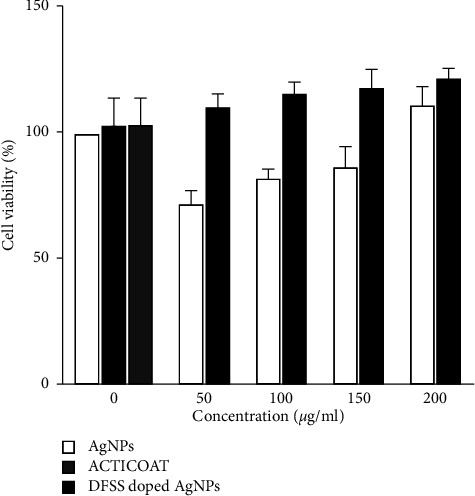
Cell viability at different concentrations of mouse fibroblast 3T3 cells obtained using the MTT assay after incubation in DFS scaffold, AgNPs, Acticoat, and DFS scaffold doped with AgNPs for 48 hours. Each value represents the mean and standard deviation of the triplicate experiment.

The biocompatibility of DFS scaffold and AgNPs were evaluated using the mouse fibroblast 3T3 (MF3T3) cell line. The viability of mouse fibroblast 3T3 in the presence of test scaffold was observed using the MTT assay for a period of 48 hours, and the results obtained are given in Figure 17. The required data for calculation of cell viability % from OD value obtained from the MTT assay, ordinary two-way ANOVA analysis of the data obtained from the MTT assay, and ordinary two-way ANOVA multiple comparisons of the test samples are shown in Tables S8, S9, and S10, respectively (supporting information). The data obtained from the ELISA Reader were analyzed using GraphPad Prism 9.2.0. Significant differences between specimens were evaluated using two-way ANOVA.

The values obtained from the analysis of the MTT assay indicated that the DFS scaffold doped with different concentrations of AgNPs showed no significant difference from the control treatment (*p* < 0.05). However, the developed scaffold helped in the proliferation of fibroblast cells. When the cells were seeded on the DFS scaffold doped with AgNPs, MF3T3 cells attached and proliferated well, as shown by the increased metabolic activity over 48 hours in the MTT assay. In tissue engineering, one of the main requirements of a scaffold is biocompatibility, which is the ability to support normal cellular activity without any toxic effects on the host tissue. The developed scaffold created a good ambience for the cell to proliferate without showing any toxicity to the cell.

AgNPs also showed the viability of MF3T3 cells, but the result was significantly less than the DFS scaffold (*p* < 0.05). This result indicates that the silver nanoparticle has cell proliferation properties. The cell proliferation of silver nanoparticles loaded with DFS scaffold increases significantly. The reason behind this might be due to the DFS scaffold which provides a better environment for the cells to attach and proliferate than AgNPs alone. Furthermore, the viability of cells has been increased with an increase in the concentration of AgNPs. However, there is no significant increase in the viability of cells when the concentration of loaded silver nanoparticles is above 50 *μ*g/ml in the DFS scaffold. This shows that a minimum concentration of 50 *μ*g/ml of AgNPs will be enough for the cell to proliferate when loaded on the DFS scaffold. Also, from the antimicrobial assay, it was seen that the concentration of 50 *μ*g/ml of AgNPs was enough to inhibit bacterial growth. Thus, a concentration of 50 *μ*g/ml AgNPs will be the appropriate concentration to be loaded on the scaffold considering the relation between the release rate of nanoparticles and the area of the scaffold.

For a wound to heal ideally, the healing agent must create a moisture environment, reduce infection, mimic the extracellular matrix feature, and reduce the wound scar [75]. During the inflammatory phase which is the initial phase of the wound healing process, cells such as neutrophils and monocytes travel to the wound site to prevent probable infection. Silver nanoparticles become more active in this phase, as it acts as an antibacterial medium and helps prevent potential bacterial growth and infection. AgNPs also activate macrophages and the immune cell [76]. Nanoparticles loaded with DFS scaffold will act as a barrier to the outer environment. The inflammatory phase is followed by the proliferative phase where keratinocytes and fibroblasts are activated which assist in closure and restoration of the vascular network. In this phase, both AgNPs and DFS scaffold will be active; DFS scaffold creates a 3D structure for the fibroblast and keratinocytes to attach and proliferate. Also, AgNPs help in acceleration of fibroblast migration [76]. Similarly, AgNPs themselves have the wound healing property; bacterial synthesis of AgNPs using *Bacillus cereus* and *Escherichia fergusonii* showed accelerated formation of collagen and epithelization [77]. It is described in the literature that silver nanoparticles can modulate anti-inflammatory cytokine release and promote rapid wound closure without increasing scarring. They can also promote epidermal re-epithelization by causing keratinocyte proliferation. The moist environment of the DFS scaffold helps in angiogenesis which is also a part of the proliferative phase. The prepared scaffold does not require any dressing due to its biodegradable property. The scaffold has been shown to degrade partially within 7 days, while the scaffold takes 30 days to degrade fully.

Considering the mentioned features, the present DFS scaffold doped with AgNPs act as a promising biocompatible, wound healing agent. This concludes that the DFS scaffold doped with AgNPs is a biocompatible antimicrobial scaffold capable of enhancing the burn wound healing process.

## 4. Conclusions

A novel scaffold with antibacterial activity was successfully prepared using decellularized fish skin scaffold and biosynthesized silver nanoparticles. The FT-IR analysis of biosynthesized AgNPs concluded that some of the biological molecules of leaf are responsible for biotransformation of silver ions to AgNPs. The XRD analysis elucidated that the crystallite size of biosynthesized AgNPs ranged between 29 nm and 35 nm. The composite scaffold contains a large amount of collagen type I that increases migration and proliferation which makes it an appropriate ECM-based decellularized scaffold for tissue engineering and burn wound dressing. Moreover, the prominent characteristics of the composite scaffold, such as encompassing high collagen content, appropriate antibacterial activity, a suitable microbial barrier, good flexibility, moisture content, swelling ratio, biodegradability, and biocompatibility with ease of formulation, make the proposed DFS scaffold a suitable dressing material for burn wound healing. The results obtained from the *in vitro* experiments met the expectations.

## Data Availability

The data used to support the findings of this study are available within its supplementary materials.

## Supplementary Materials

Supplementary Materials XRD analysis data of peak position, full-width-half-maximum (FWHM), calculation of size, and average size of both AgNPs samples; DPPH radical scavenging activity (%RSA) and IC50 value of *Aloe vera* extract, biosynthesized and chemically synthesized silver nanoparticles, and ascorbic acid (standard); percentage weight loss of the DFS scaffold during in vitro degradation at 37°C in PBS solution, having pH 7.4; percentage of swelling ratio or water uptake capacity of DFS scaffold at 1, 2, 3, 12, and 24 hours; percentage moisture content of DFS scaffold; determination of nanoparticle loading capacity of DFS scaffold by dipping in different concentrations of AgNPs solutions; the AgNP release profile from DFS scaffold at different time intervals; ordinary two-way ANOVA analysis of the data obtained from the MTT assay; extracted *Aloe vera* which was used for the preparation of AgNPs in the hydrothermal method; samples of purified AgNPs prepared in 6 h and 12 h; photographs of adult tilapia fish, extraction of skin tissue from tilapia fish, and decellularized skin scaffold used in research; figure of the antioxidant assay of biosynthesized AgNPs and *Aloe vera* extract compared with chemically synthesized AgNPs and ascorbic acid; samples of dried DFS scaffold in desiccators after the swelling test.
