# Supplementary material for: Development of Decellularized Fish Skin Scaffold Decorated with Biosynthesized Silver Nanoparticles for Accelerated Burn Wound Healing
Source: Int J Biomater. 2023 Jan 31;2023:8541621. doi: 10.1155/2023/8541621 (PMC9904935; doi:10.1155/2023/8541621)
Supplement: Supplementary Materials — XRD analysis data of peak position, full-width-half-maximum (FWHM), calculation of size, and average size of both AgNPs samples; DPPH radical scavenging activity (%RSA) and IC50 value of Aloe vera extract, biosynthesized and chemically synthesized silver nanoparticles, and ascorbic acid (standard); percentage weight loss of the DFS scaffold during in vitro degradation at 37°C in PBS solution, having pH 7.4; percentage of swelling ratio or water uptake capacity of DFS scaffold at 1, 2, 3, 12, and 24 hours; percentage moisture content of DFS scaffold; determination of nanoparticle loading capacity of DFS scaffold by dipping in different concentrations of AgNPs solutions; the AgNP release profile from DFS scaffold at different time intervals; ordinary two-way ANOVA analysis of the data obtained from the MTT assay; extracted Aloe vera which was used for the preparation of AgNPs in the hydrothermal method; samples of purified AgNPs prepared in 6 h and 12 h; photographs of adult tilapia fish, extraction of skin tissue from tilapia fish, and decellularized skin scaffold used in research; figure of the antioxidant assay of biosynthesized AgNPs and Aloe vera extract compared with chemically synthesized AgNPs and ascorbic acid; samples of dried DFS scaffold in desiccators after the swelling test. [file 8541621.f1.docx]

**Development of decellularized fish skin scaffold decorated with biosynthesized silver nanoparticles for accelerated burn wound healing**

Surya Prasad Adhikari^1, 2*^, Anisha Sharma^2^, Astha Paudel^2^, Baruna Thapa^2^, Neha Khanal^2^, Nisha Shastri^2^, Sourav Rai^2^, Rameshwar Adhikari ^3,^ *

^1^Department of Mechanical and Aerospace Engineering, Pulchowk Campus, IOE, TU, Nepal

^2^College of Biomedical Engineering and Applied Sciences, Purwanchal University, Nepal

^3^Centre Department of Chemistry, TU, Nepal

^*^Corresponding Authors

**Supporting Data**

Table S1: XRD analysis data showing peak position, Full-Width-Half-Maximum (FWHM), size and average size of sample 1 and 2 AgNPs.

| Sample | Peak position (2θ) | FWHM (β) | Size (nm) | Average size (nm) |
| --- | --- | --- | --- | --- |
| 1 | 32.654 | 0.285 | 28.387 | 29.612 |
|  | 46.627 | 0.285 | 29.620 |  |
|  | 28.237 | 0.271 | 29.519 |  |
|  | 55.208 | 0.283 | 30.923 |  |
| 2 | 32.758 | 0.219 | 37.770 | 35.203 |
|  | 28.347 | 0.226 | 36.103 |  |
|  | 46.735 | 0.247 | 34.933 |  |
|  | 57.950 | 0.283 | 32.005 |  |

Table S2: DPPH radical scavenging activity (%RSA) and IC50 value of aloevera extract, biosynthesized silver nanoparticles using aloe vera leaf extract, chemically synthesized silver nanoparticles, ascorbic acid (standard).

| Control absorbance | | 0.301 | | | | | |
| --- | --- | --- | --- | --- | --- | --- | --- |
| Treatments | Concentration(µg) | Sample1 | Sample2 | Sample3 | Average | % RSA | IC50  value |
| Aloe veraextract | 25 | 0.274 | 0.285 | 0.294 | 0.284 | 5.537 | 307 |
|  | 50 | 0.246 | 0.247 | 0.256 | 0.250 | 17.054 |  |
|  | 100 | 0.223 | 0.236 | 0.227 | 0.229 | 24.031 |  |
|  | 200 | 0.204 | 0.207 | 0.194 | 0.202 | 33.001 |  |
| Green synthesis of AgNps | 25 | 0.295 | 0.304 | 0.303 | 0.301 | 0.111 | 362 |
|  | 50 | 0.283 | 0.292 | 0.249 | 0.275 | 8.749 |  |
|  | 100 | 0.225 | 0.258 | 0.275 | 0.253 | 16.058 |  |
|  | 200 | 0.229 | 0.227 | 0.211 | 0.222 | 26.135 |  |
| Chemical synthesis of AgNps | 25 | 0.285 | 0.272 | 0.282 | 0.280 | 7.087 | 620 |
|  | 50 | 0.267 | 0.262 | 0.276 | 0.268 | 10.853 |  |
|  | 100 | 0.263 | 0.255 | 0.282 | 0.267 | 11.406 |  |
|  | 200 | 0.283 | 0.223 | 0.243 | 0.250 | 17.054 |  |
| Ascorbic acid (standard) | 25 | 0.082 | 0.086 | 0.083 | 0.084 | 56.873 | 7.402 |
|  | 50 | 0.082 | 0.079 | 0.088 | 0.083 | 57.216 |  |
|  | 100 | 0.072 | 0.062 | 0.070 | 0.068 | 64.948 |  |
|  | 200 | 0.042 | 0.047 | 0.048 | 0.046 | 76.460 |  |

Table S3: Percentage weight loss of the DFS scaffold during in-vitro degradation at 37^o^C in PBS solution, pH 7.4.

| Time (day) | Weights (gm) | Degradation rate (%) | Mean (%) | Std. |
| --- | --- | --- | --- | --- |
| 0 | 0.220 | 0 | 0 | 0 |
|  | 0.125 | 0 |  |  |
|  | 0.127 | 0 |  |  |
| 1 | 0.160 | 27.273 | 19.420 | 7.120 |
|  | 0.103 | 17.600 |  |  |
|  | 0.110 | 13.386 |  |  |
| 2 | 0.140 | 36.364 | 29.074 | 7.566 |
|  | 0.088 | 29.600 |  |  |
|  | 0.100 | 21.260 |  |  |
| 3 | 0.134 | 39.091 | 33.425 | 6.731 |
|  | 0.081 | 35.200 |  |  |
|  | 0.094 | 25.984 |  |  |
| 4 | 0.123 | 44.091 | 40.379 | 5.037 |
|  | 0.072 | 42.400 |  |  |
|  | 0.083 | 34.646 |  |  |
| 5 | 0.115 | 47.727 | 43.441 | 5.104 |
|  | 0.069 | 44.800 |  |  |
|  | 0.079 | 37.795 |  |  |
| 6 | 0.110 | 50.000 | 47.898 | 3.300 |
|  | 0.063 | 49.600 |  |  |
|  | 0.071 | 44.094 |  |  |
| 7 | 0.099 | 55.000 | 50.627 | 5.197 |
|  | 0.060 | 52.000 |  |  |
|  | 0.070 | 44.882 |  |  |
| 14 | 0.083 | 62.273 | 59.655 |  |
|  | 0.050 | 60.000 |  |  |
|  | 0.055 | 56.693 |  |  |
| 21 | 0.063 | 71.364 | 68.231 | 2.726 |
|  | 0.042 | 66.400 |  |  |
|  | 0.042 | 66.929 |  |  |

Table S4: Swelling ratio or water uptake capacity of DFS scaffold. The swelling ratio % of DFS scaffold at PBS solution was found to be 74.7%, 89.5%, 96.86%, 98.45% and 102.94% at 1, 2, 3, 12 and 24 hours respectively.

| Time(hr) | Weights(gm) | Swellingratio | Swelling percent(%) | Mean | St.d |
| --- | --- | --- | --- | --- | --- |
| 0 | 0.105 | - | - | - | - |
|  | 0.146 | - | - |  |  |
|  | 0.132 | - | - |  |  |
| 1 | 0.173 | 0.648 | 64.76 | 74.669 | 9.746539021 |
|  | 0.269 | 0.842 | 84.25 |  |  |
|  | 0.231 | 0.750 | 75.00 |  |  |
| 2 | 0.193 | 0.838 | 83.81 | 89.577 | 8.48121805 |
|  | 0.291 | 0.993 | 99.32 |  |  |
|  | 0.245 | 0.856 | 85.61 |  |  |
| 3 | 0.204 | 0.943 | 94.29 | 96.857 | 2.516576192 |
|  | 0.291 | 0.993 | 99.32 |  |  |
|  | 0.260 | 0.970 | 96.97 |  |  |
| 12 | 0.206 | 0.962 | 96.19 | 98.45 | 2.247392494 |
|  | 0.293 | 1.007 | 100.68 |  |  |
|  | 0.262 | 0.985 | 98.48 |  |  |
| 24 | 0.210 | 1.000 | 100.00 | 102.89 | 2.507982518 |
|  | 0.298 | 1.041 | 104.11 |  |  |
|  | 0.270 | 1.045 | 104.55 |  |  |

Table S5: Moisture content of DFS scaffold. The moisture content of DFS scaffold was found to be 81.7±3.6%.

| Sample | Initial Weight (gm) | Final weight (gm) | Moisture content % |
| --- | --- | --- | --- |
| DFSS1 | 0.191 | 0.033 | 82.723 |
| DFSS2 | 0.239 | 0.038 | 84.100 |
| DFSS3 | 0.276 | 0.041 | 85.145 |
| DFSS4 | 0.283 | 0.046 | 83.746 |
| DFSS5 | 0.239 | 0.056 | 76.569 |
| DFSS6 | 0.190 | 0.042 | 77.895 |

Table S6: Determination of AgNPs loading capacity on DFS Scaffold. The loading capacity of DFS dopped in 150µg/ml and 200µg/ml AgNPs were found to be 48.7µg/mm^2^ and 61.5µg/mm^2^ respectively.

| Concentration | Sample | Absorbance before doping (X) | Absorbance after doping (Y) | Doped  % [  (X-Y)  *100/X] | Area of scaffold (c㎡) | Doped concentration per area (μg/cm^2^) | Mean | St.d |
| --- | --- | --- | --- | --- | --- | --- | --- | --- |
| 150 | 1 | 0.22500 | 0.14600 | 35.111  11 | 1.130  97 | 46.56755  742 | 38.  71 | 12.  31 |
|  | 2 | 0.21200 | 0.14000 | 33.962  26 | 1.130  97 | 45.04385  182 |  |  |
|  | 3 | 0.23800 | 0.19400 | 18.487  39 | 1.130  97 | 24.51966  91 |  |  |
| 200 | 1 | 0.25400 | 0.17400 | 31.496  06 | 1.130  97 | 55.69726  792 | 62.  74 | 7.3  29 |
|  | 2 | 0.25900 | 0.15600 | 39.768  34 | 1.130  97 | 70.32586  503 |  |  |
|  | 3 | 0.25300 | 0.16400 | 35.177  87 | 1.130  97 | 62.20812  444 |  |  |

Table S7: AgNPs release profile from DFS scaffold at different time.

| Concentration (µg) | 150 | | | | | 200 | | | | |
| --- | --- | --- | --- | --- | --- | --- | --- | --- | --- | --- |
| sample | 1 | 2 | 3 | Mea n | St.d | 1 | 2 | 3 | Mean | St.d |
| absorbance at 2 hrs | 0.08  2 | 0.08  1 | 0.07  9 | 0.08  1 | 0.001  5 | 0.07  5 | 0.08  7 | 0.05  9 | 0.07  4 | 0.014 |
| absorbance at 4 hrs | 0.09 | 0.08  5 | 0.08  2 | 0.08  6 | 0.004 | 0.07  5 | 0.07  7 | 0.06  9 | 0.07  3 | 0.004  2 |
| absorbance at 24 hrs | 0.12  1 | 0.12  1 | 0.11  3 | 0.09  4 | 0.004  6 | 0.09  6 | 0.09 | 0.09  6 | 0.09  4 | 0.003  5 |
| significant | yes | yes | yes |  |  | yes | yes | yes |  |  |

Table S8: Calculation of cell viability % from OD value obtained from MTT assay.

| Treatments | | OD Value | | | % Cell Viability (%CV) | | | Mean of  %CV | Standarddeviation of  %CV |
| --- | --- | --- | --- | --- | --- | --- | --- | --- | --- |
|  |  | 1 | 2 | 3 | 1 | 2 | 3 |  |  |
| control | | 0.10  9 | 0.10  7 | 0.11  4 | 100 | 100 | 100 | 100 | 0 |
| ACTicoat | | 0.12  1 | 0.11  5 | 0.10  4 | 111.0  1 | 107.4  8 | 91.23 | 103.2  4 | 10.55 |
| DFSS | | 0.10  3 | 0.12  3 | 0.11  3 | 94.50 | 114.9  5 | 99.12 | 102.8  6 | 10.73 |
| AgNPs only | 50  μg/ml | 0.07  5 | 0.08  3 | 0.07  9 | 68.81 | 77.57 | 69.30 | 71.89 | 4.92 |
|  | 100  μg/ml | 0.09  0 | 0.09  1 | 0.08  8 | 82.57 | 85.05 | 77.19 | 81.60 | 4.01 |
|  | 150  μg/ml | 0.09  3 | 0.08  5 | 0.10  8 | 85.32 | 79.44 | 94.74 | 86.50 | 7.72 |
|  | 200  μg/ml | 0.13  0 | 0.11  5 | 0.12  0 | 119.2  7 | 107.4  8 | 105.2  6 | 110.6  7 | 7.53 |
| DFSS  dopedAgNP  s | 50  μg/ml | 0.12  2 | 0.11  1 | 0.13  0 | 111.9  3 | 103.7  4 | 114.0  4 | 109.9  0 | 5.44 |
|  | 100  μg/ml | 0.12  5 | 0.12  9 | 0.12  7 | 114.6  8 | 120.5  6 | 111.4  0 | 115.5  5 | 4.64 |
|  | 150  μg/ml | 0.12  3 | 0.13  5 | 0.13  0 | 112.8  4 | 126.1  7 | 114.0  4 | 117.6  8 | 7.37 |
|  | 200 | 0.13 | 0.13 | 0.13 | 120.1 | 126.1 | 118.4 | 121.5 | 4.06 |
|  | μg/ml | 1 | 5 | 5 | 8 | 7 | 2 | 9 |  |

Table S9: Ordinary Two-way anova analysis of the data obtained from MTT assay.

| Two-wayANOVA | Ordinary |  |  |  |  |
| --- | --- | --- | --- | --- | --- |
| Alpha | 0.05 |  |  |  |  |
| SourceofVariation | % of totalvariation | P  value | P valuesummary | Significant? |  |
| Concentrations | 23.44 | 0.002  9 | ** | Yes |  |
| Treatments | 47.93 | <0.00  01 | **** | Yes |  |
| ANOVAtable | SS (TypeIII) | DF | MS | F (DFn, DFd) | P  value |
| Concentrations | 2009 | 4 | 502.2 | F (4,26) =  5.320 | P=0.0029 |
| Treatments | 4107 | 2 | 2054 | F (2,26) =  21.75 | P<0.0001 |
| Residual | 2454 | 26 | 94.40 |  |  |
| Data summary |  |  |  |  |  |
| Number of columns (Treatments) | 3 |  |  |  |  |
| Number of rows (Concentrations) | 5 |  |  |  |  |
| Number of values | 33 |  |  |  |  |

Table S10: Ordinary two-way anova multiple comparisons of the test samples.

| Comparecellmeansregardlessofrowsandcolumns |  |  |  |  |  |
| --- | --- | --- | --- | --- | --- |
| Numberoffamilies | 1 |  |  |  |  |
| Numberofcomparisonsperfamily | 55 |  |  |  |  |
| Alpha | 0.05 |  |  |  |  |
| Tukey's multiplecomparisonstest | Predicted (LS) mean diff. | 95.00%  CI of  diff. | Belowthreshol d? | Summary | AdjustedPValue |
| 0: AgNPs vs. 0: DFSS  Doped AgNPs | -23.38 | -36.62to  -10.14 | Yes | **** | <0.0001 |
| 0: AgNPs vs. 0:ACTICOAT | -13.50 | -39.98to  12.97 | No | ns | 0.8316 |
| 0:AgNPs vs. 50: AgNPs | 10.53 | -10.40to  31.46 | No | ns | 0.8439 |
| 0:AgNPsvs. 50: DFSS  Doped AgNPs | -12.85 | -37.62to  11.91 | No | ns | 0.8149 |

**Supporting figure:**


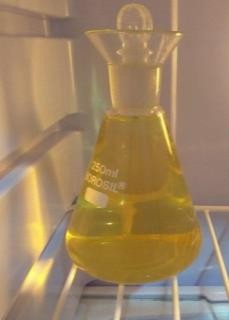


Figure S1: Aloe vera extract which was used for the preparation of AgNPs using hydrothermal method.


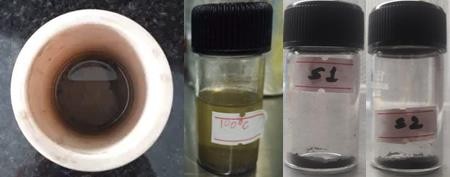


Figure S2: Silver nanoparticles prepared at 100^o^C varying incubation time condition (i.e., 6 hrs and 12 hrs) (a) Teflon lined vessel, (b) reduction of clear AgNO_3_ to brown colour solution by aloevera extract which will be incubated at 100ºC for 6 hrs and 12 hrs respectively, (c)&(d) purified and dried AgNPs to obtained powdered AgNPs.


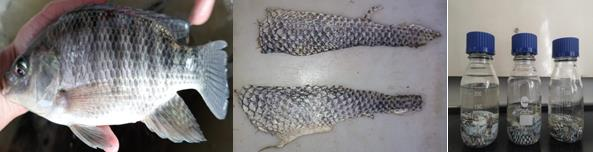


Figure S3: Decellularization of Tilapia fish skin. (a) adult Tilapia fish, (b) extraction skin tissue from tilapia fish and (c) decellularized fish skin scaffold.


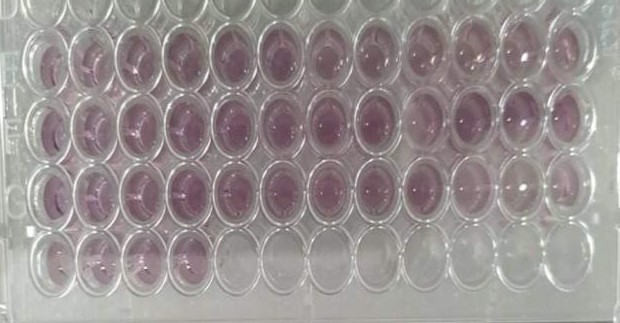


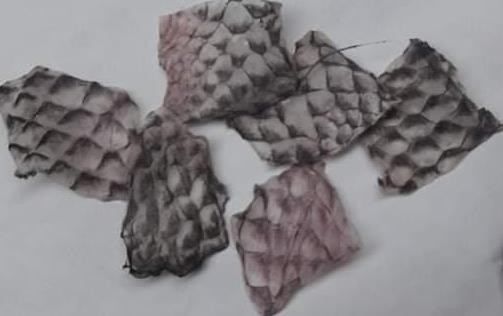
Figure S4: Antioxidant assay of biosynthesized AgNPs and Aloe vera extract compared with chemically synthesized AgNPs and Ascorbic acid.

Figure S5: Dried DFS scaffold in desiccators after swelling test.
